# The Radiated Deep-frozen Xenogenic Meniscal Tissue Regenerated the Total Meniscus with Chondroprotection

**DOI:** 10.1038/s41598-018-27016-w

**Published:** 2018-06-13

**Authors:** Dong Jiang, Zheng-Zheng Zhang, Feng Zhao, Shao-Jie Wang, Yan-Song Qi, Li-Heng Zhao, Ji-Ying Zhang, Jia-Kuo Yu

**Affiliations:** 1 Institute of Sports Medicine, Beijing Key Laboratory of Sports Injuries, Peking University Third Hospital, Beijing, 100191 P. R. China; 2 Department of Orthopaedics, Sun Yat-sen Memorial Hospital, Sun Yat-sen University, Guangzhou, 510120 P. R. China; 3 Key Laboratory for Biomechanics and Mechanobiology of Ministry of Education, School of Biological Science and Medical Engineering, Beihang University, Beijing, 100191 P. R. China; 4 Department of Joint Surgery, Zhongshan Hospital of Xiamen University, Xiamen University, Xiamen, 361004 P. R. China

## Abstract

Meniscal allograft transplantation yields good and excellent results but is limited by donor availability. The purpose of the study was to evaluate the effectiveness of radiated deep-frozen xenogenic meniscal tissue (RDF-X) as an alternative graft choice in meniscal transplantation. The xenogenic meniscal tissues were harvested from the inner 1/3 part of the porcine meniscus and then irradiated and deeply frozen. The medial menisci of rabbits were replaced by the RDF-X. Meniscal allograft transplantation, meniscectomy and sham operation served as controls. Only a particular kind of rabbit-anti-pig antibody (molecular ranging 60–80 kD) was detected in the blood serum at week 2. The menisci of the group RDF-X grossly resembled the native tissue and the allograft meniscus with fibrocartilage regeneration at postoperative 1 year. Cell incorporation and the extracellular matrix were mostly observed at the surface and the inner 1/3 part of the newly regenerated RDF-X, which was different from the allograft. The biomechanical properties of the group RDF-X were also approximate to those of the native meniscus except for the compressive creep. In addition, chondroprotection was achieved after the RDF-X transplantation although the joint degeneration was not completely prevented. To conclude, the RDF-X could be a promising alternative for meniscal transplantation with similar tissue regeneration capacity to allograft transplantation and superior chondroprotection. The potential minor immunological rejection should be further studied before its clinical application.

## Introduction

Meniscus plays an important role in stabilization and lubrication of the knee joint. Meniscus replacement aims to re-establish normal joint load transmission to prevent the degeneration of the articular cartilage after meniscectomy^1,2^. Meniscus allograft transplantation has been proven promising for pain relief^3^ and Noyes *et al*. evaluated the long-term results of meniscus transplantation with successful outcome. However, joint degeneration could not be completely prevented by meniscus transplantation^4,5^. Moreover, the availability of allograft is very limited and there are concerns over the possibility of disease transmission^6,7^.

The Collagen Meniscus Implants (CMI) is a structure fabricated by collagen type I isolated and purified from bovine Achilles tendon. This normal human meniscus sized implant is enriched with glycosaminoglycans (GAGs). Rodkey *et al*.^8^ reported the results of implantation of CMI at 2-year follow-up. All patients have exhibited clinical symptoms improvement and the regenerated tissue showed new fibrocartilage tissue formation. Steadman^9^ and Zaffagnini *et al*.^10^ reported the results of CMI implantations with more than five years follow-up, respectively. Both studies shed light on its promising clinical evolution and assumed chondroprotection. However, the transplanted CMI appeared shrinkage in the study with larger population^11^.

Xenografts, such as porcine small intestinal submucosa (SIS), have been used to replace meniscal tissue in a dog model^12^. However, the biochemical and biomechanical properties of SIS could not perfectly match those of the native meniscus, thus the clinical translation have not been reported^13^. In this study, porcine menisci was treated with irradiation and deep freezing, and then implanted into rabbit knee joints. Compared with other methods of decellularization^14^, irradiation and deep freezing was more simple and efficient, which have been applied for meniscus allograft processing. The purpose of the present study was to evaluate the potential of the radiated deep-frozen xenogenic meniscal tissue (RDF-X) as alternative for meniscal transplantation. Tissue regeneration and chondroprotective effects of RDF-X (Xeno group) were evaluated at one year follow-up, compared with those of the allograft (Allo group), meniscectomy (Meni group), or the sham surgery (Sham group). We hypothesized that the RDF-X could bring about certain benefits in meniscal regeneration and long-term protection of the articular cartilage. There might be difference between the xenograft group and the allograft group in terms of cell infiltration and tissue regeneration.

## Results

### Characterization of the RDF-X meniscal tissue

**Figure 1:**
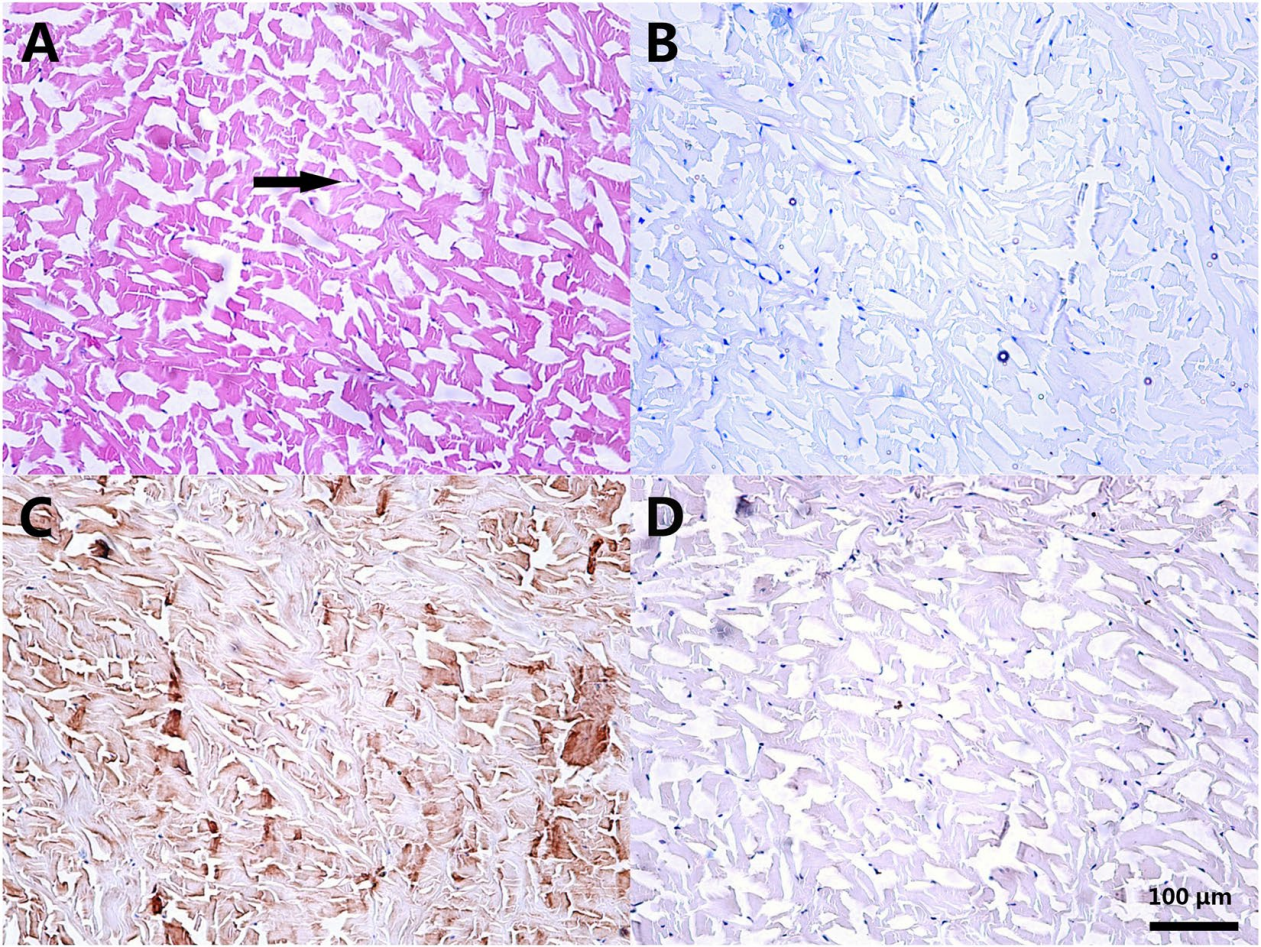
Characterization of the radiated deep-frozen xenogenic meniscal tissue (RDF-X) meniscal tissue. (**A**) The collagen network collapsed and originally exsisted fibroacartilage cells appeared karyopyknosis (black arrow). The glycosaminoglycans and collagen type I and II (Col I and II) were markedly reduced after the process throughout the toluidine blue (TB) and immunohistochemistry (IHC) for Col I and II staining (**B**–**D**). Scale bar = 100 μm.

As shown in Fig. 1, the collagen network collapsed and the dense internal structure were damaged. A large amount of gaps were found between the loose collagen fibers. The originally existed fibrocartilage cells were extinct and appeared karyopyknosis. According to the toluidine blue (TB) staining results, the GAGs obviously reduced after the treatment and the original neatly arranged fiber became messy. The significantly lighter color was shown in the immunohistochemistry (IHC) for the collagen type I (Col I) and the collagen type II (Col II).

Compared to the native meniscus in scanning electron microscope (SEM), collagen bundles in the RDF-X appeared to be looser. Gaps and micropores were found within the destructive collagen network (approximately 20–150 μm).

### Assessment of immunological rejection

**Figure 2:**
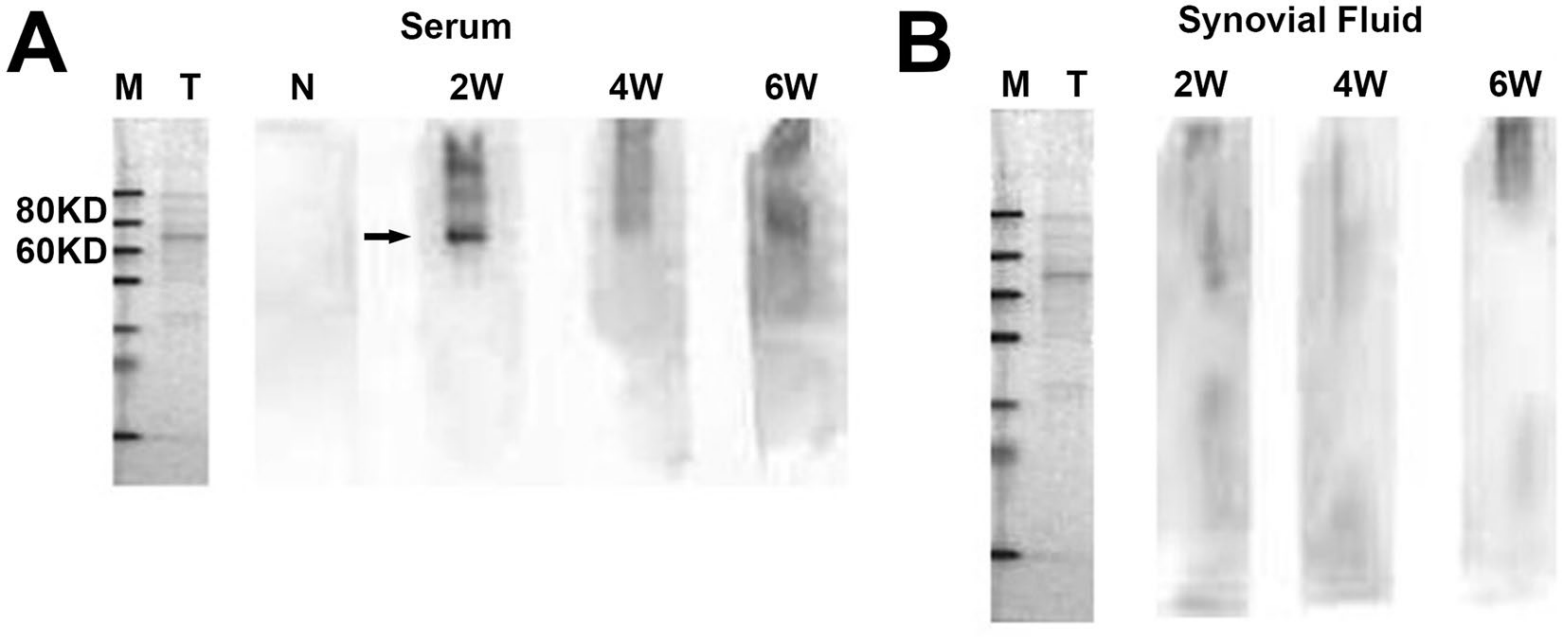
Immunological rejection assayed in Western-blot. (**A**) A kind of rabbit-anti-pig antibody of the molecular ranging 60–80kD was detected at week 2 in the blood serum (black arrow). No antibody was detected in the joint fluid from postoperative week 2 to week 6 (**B**).

According to the result of the Western-blot (Fig. 2), no antibody was detected in the joint fluid from postoperative week 2 to week 6. In the blood serum, a rabbit-anti-pig antibody with the molecular ranging 60–80 kD was detected at week 2. However, this antibody was not detected at week 4 and week 6.

### Gross evaluation of the meniscus

**Figure 3:**
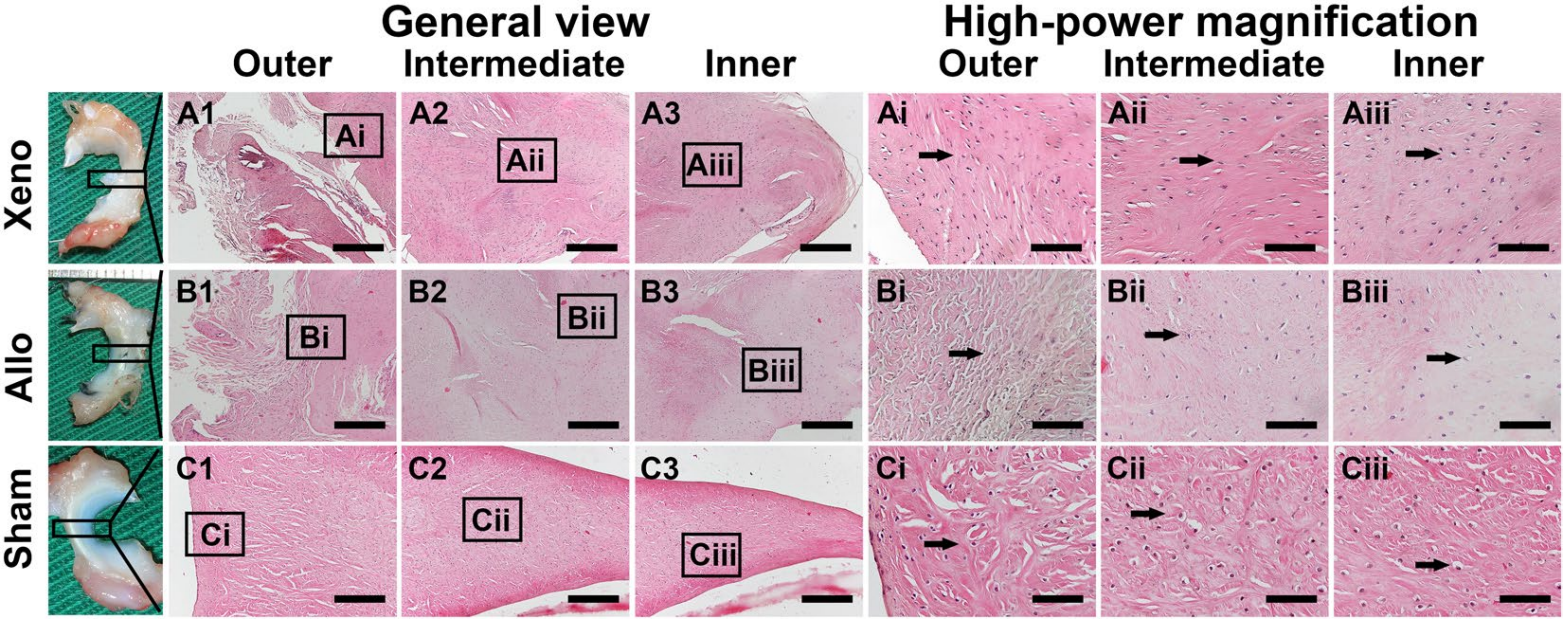
Gross and historical (general view and high-power magnification) images of meniscus tissue. Macroscopic view of implants or native menisci are show on the left. Insert box identifies the region analyzed by histology as listed below. General view of outer, intermediate, and inner zone of implants or native menisci (hematoxylin and eosin (H&E) staining) are show in the middle (Scale bar = 100 μm). High-power magnification images of meniscus tissue are shown on the right (Scale bar = 25 μm). Integrated cells (black arrow) were observed in the implants of Xeno group and Allo group.

One rabbit of the Allo group died of diarrhea at week 30 and was excluded. Ten menisci of the Xeno group and 9 menisci of the Allo group were obtained at postoperative 1 year for evaluation (Fig. 3). All the grafts healed to their normal attachment sites, with no sign of disruption or gap formation. Seven menisci in the Xeno group seemed approximately normal at gross glance with a shiny-white color and a smooth surface. No obvious synovium hypertrophy was detected. Meniscus partial tear with rough surface was found in three menisci of the Xeno group. In the Allo group, all the menisci remained normal except for one with mild shrinkage. Calcification was found at the anterior horn of 2 menisci in each group. As shown in Table 1, the scores of the Xeno group in terms of the shape, tears, surface and tissue were significantly lower than those of the Allo group (*P* < 0.05). However, no significant difference was found in the total scores.

**Table 1:** Gross evaluation of meniscus implant score.

	Xeno Group	Allo Group	*P* Values
Integration	2.0 ± 0.3	2.8 ± 0.4	0.658
Implant position	2.2 ± 0.6	2.5 ± 0.7	0.242
Horn position	2.9 ± 0.3	2.5 ± 0.7	0.098
Shape	2.2 ± 0.8	2.9 ± 0.3	0.01*
Tears	2.1 ± 0.9	2.9 ± 0.3	0.009*
Surface	2.2 ± 0.6	2.8 ± 0.4	0.012*
Size	2.3 ± 0.5	2.4 ± 0.5	0.58
Tissue	2.3 ± 0.5	2.8 ± 0.4	0.013*
Synovial	1.6 ± 0.5	2.0 ± 0.4	0.062
Total score	20.7 ± 3.5	23.8 ± 3.1	0.053

*Statistically significant (*P* < 0.05).

### Histology of the meniscus

The synovial cells infiltrated into the graft over time in most of the transplanted menisci. The semi-quantitative histological analysis of both groups were shown in Table 2. The results of the overall parameters were similar between the two groups except for the lymphocyte infiltration. However, for the cell infiltration and the GAG distribution (Table 3), difference existed between the Xeno group and the Allo group. The relatively uniform distribution was found for the outer, intermediate and the inner 1/3 zone of the Xeno graft but most of the cells concentrated in the outer 1/3 zone of the allograft.

**Table 2:** Histological features of implants.

	Xeno Group [n (%)]	Allo Group [n (%)]
Residual scaffold	9 (90)	9 (100)
Foreign body reaction	2 (20)	0
Hypocellular areas	8 (80)	6 (66.7)
Blood vessels	9 (90)	7 (77.8)
Fibrosis	9 (90)	7 (77.8)
Cartilage metaplasia		
Tip	9 (90)	8 (88.9)
Central	5 (50)	4 (44.5)
Integration		
Good	10 (100)	8 (88.9)
Poor	0	1 (11.1)
Inflammatory infiltrate		
Lymphocytes	2 (20)	0
Plasma	0	0
Neutrophils	0	0

The table illustrated the number of implants with each of the histological features.

**Table 3:** Histological evaluation of meniscus implant in integrated cells and the TB metachromasia in each zone.

	Xeno Group	Allo Group	*P* Values
Integrated cells			
Inner	1.8 ± 1.0	1.0 ± 1.5	0.159
Intermediate	1.6 ± 1.0	1.4 ± 1.6	0.645
Outer	2.1 ± 1.5	2.4 ± 1.2	0.676
TB metachromasia			
Inner	0.8 ± 0.8	0.6 ± 0.9	0.42
Intermediate	0.9 ± 0.9	0.9 ± 0.9	0.965
Outer	1.3 ± 0.9	1.3 ± 0.7	0.893

**Figure 4:**
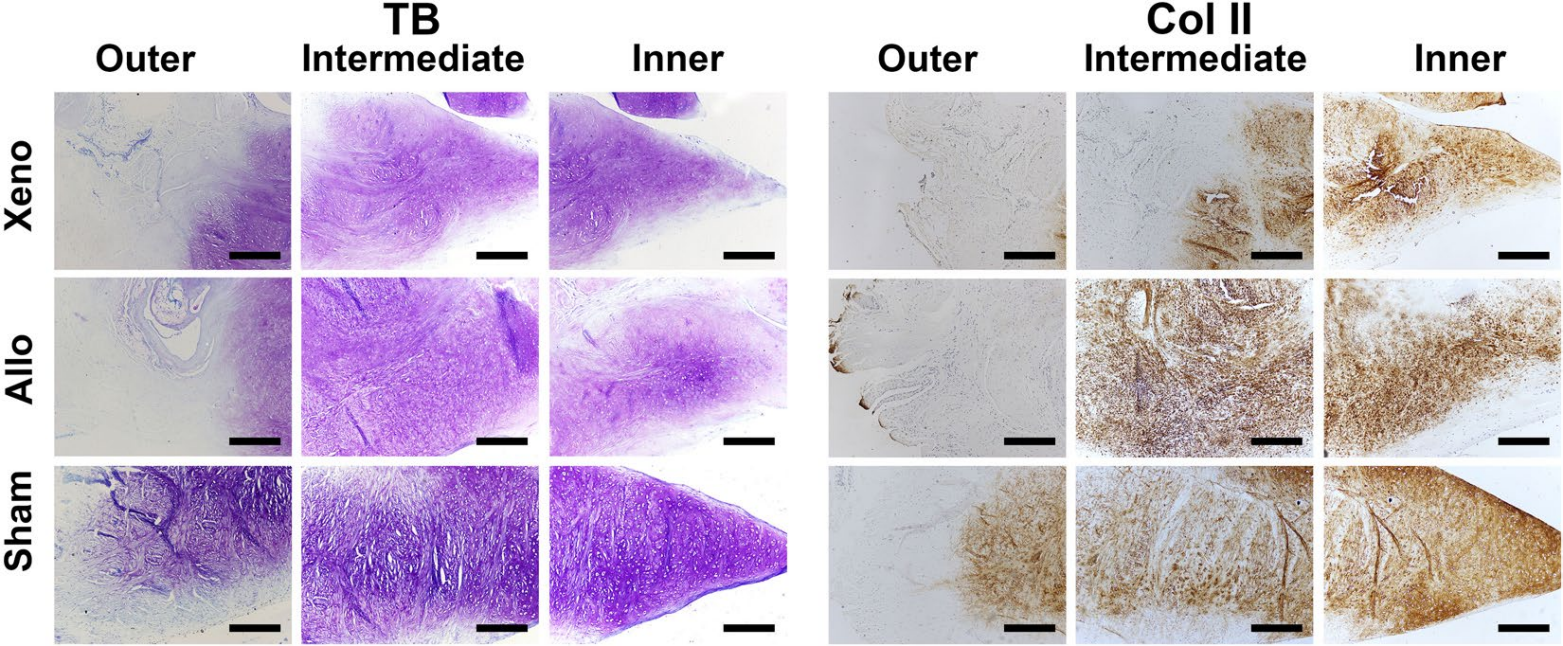
Representative TB and IHC staining for Col II of regenerated and native menisci. Xeno group showed a slighter TB staining and Col II labeling than Allo group. Scale bars represent 100 μm.

**Figure 5:**
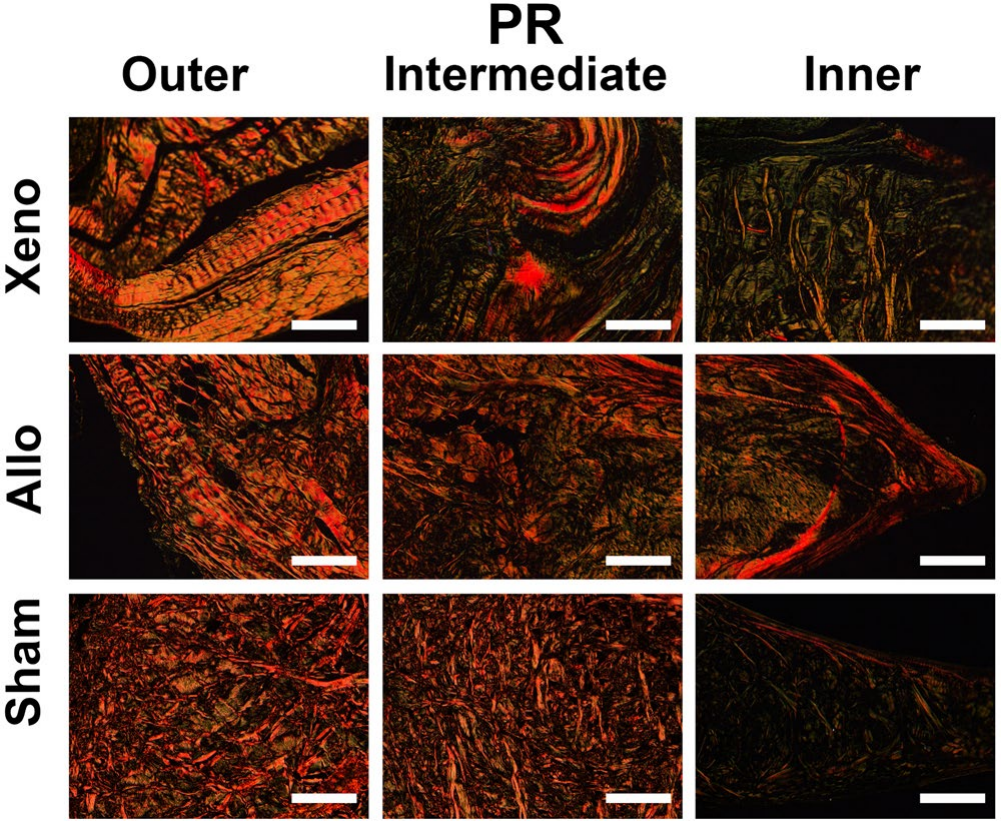
Representative picro-sirius red (PR) staining for Col I and III of regenerated and native menisci. Crassi red fiber represented Col I, and green and thin fibers with weak birefringence represented Col III. Scale bar represented 100 μm.

In the Xeno group, blood vessels and fibrous tissue were observed surrounding the graft. However, full cell incorporation and fibrocartilage regeneration were observed only in 2 menisci, which revealed no blank zone. For the other 8 menisci, cell incorporation were seen only at the upper and lower surface and the inner 1/3 part of the newly regenerated menisci. Furthermore, the phenotype of those cells was more approximate to hyaline chondrocyte surrounded by more TB staining and Col II (Fig. 4) but with few Col I and collagen type III (Col III) (Fig. 5).

In the Allo group, 2 menisci achieved full cell incorporation and demonstrated normal meniscus histology. Most of the cells concentrated in the outer 1/3 part near the synovial edge with fibrochondrocyte phenotype for 7 menisci. There was only a small amount of cells infiltration in the intermediate and the inner part of the allograft.

### Ultrastructure evaluation

**Figure 6:**
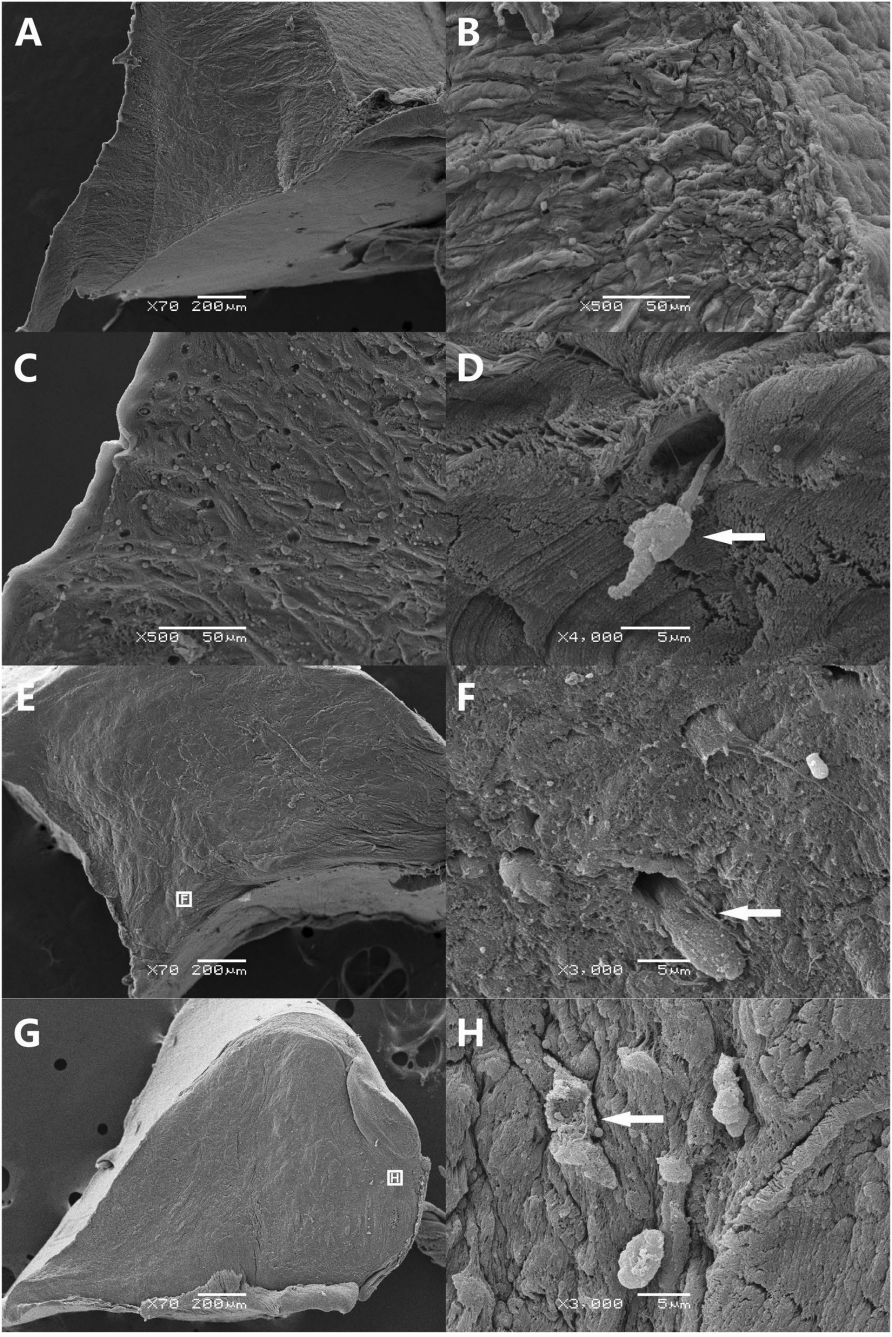
Ultrastructure evaluation of meniscus tissue. The whole (**A**), outer (**B**) and inner (**C**) regions of native rabbit meniscus. The fibrochondrocyte (white arrow) was seen in some lacunas (**D**). Menisci of the Xeno group (**E**,**F**) and the Allo group (**G**,**H**) at postoperative 1 year, and some integrated cells (white arrow) were also seen. The cell incorporation were observed only at the upper and lower surface and the inner 1/3 part of the new regenerated meniscus in the Xeno group while most of the cell infiltration concentrated in the outer 1/3 part of the graft in the Allo group.

For the RDF-X (Fig. 6E,F) and the allograft (Fig. 6G,H) at postoperative 1 year, the reconstruction of the normal structure was incomplete. However, in the areas with fibrocartilaginous tissue regeneration, the loose collagen bundle network had been replaced by the dense extracellular matrix with fibrochondrocyte in the lacunas (Fig. 6E–H), which was approximate to the native meniscus (Fig. 6A–D).

### Biomechanical evaluation

**Figure 7:**
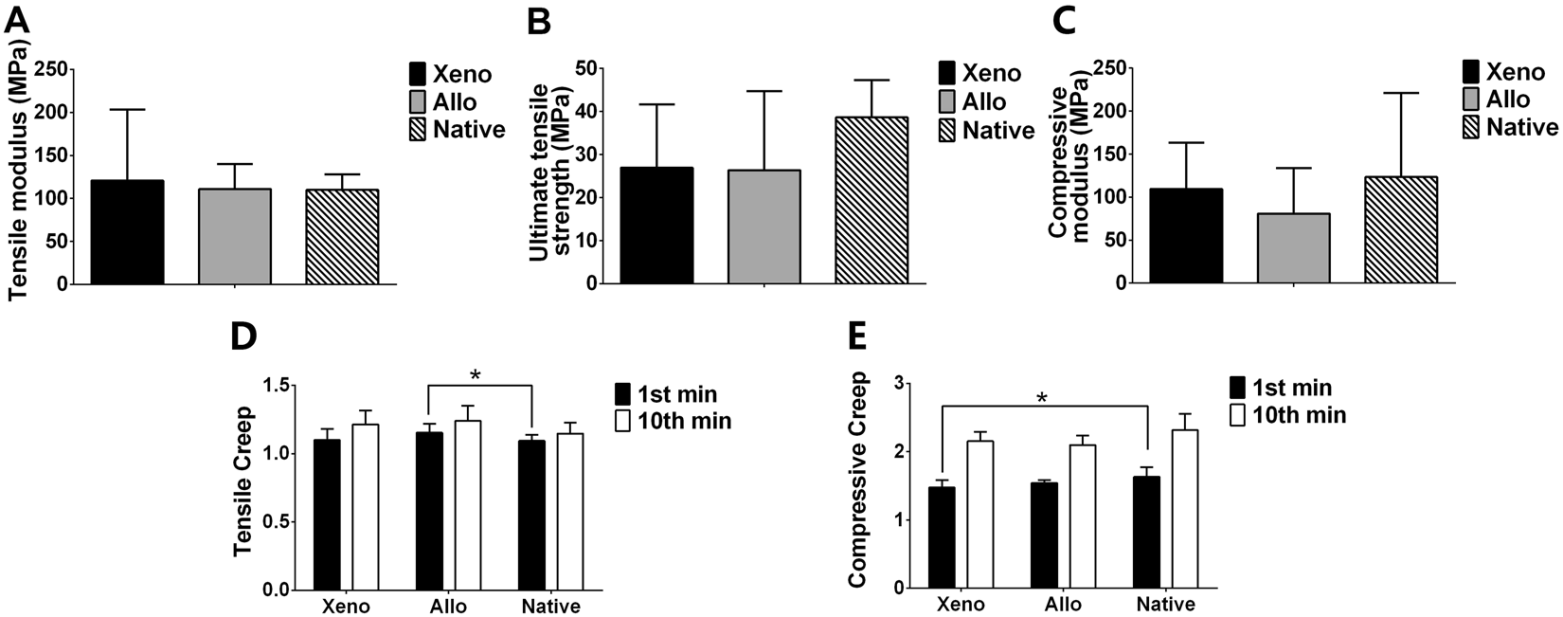
Biomechanical properties. Tensile modulus (**A**), ultimate tensile strength (**B**) and compressive modulus (**C**) of the menisci from the Xeno group and the Allo group had no significant difference compared with the native menisci. The tensile (**D**) and compressive (**E**) creep compared with the native menisci (*P < 0.05, n = 6 per group).

In Fig. 7, tensile modulus were 121.0 ± 82.2 MPa for the Xeno group, 110.9 ± 29.2 MPa for the Allo group and 109.9 ± 18.0 MPa for the Native group, respectively. No significant difference was found between the three groups (*P* > 0.05). The ultimate tensile strength of the Xeno group (27.0 ± 14.7 MPa) and the Allo group (26.39 ± 18.3 MPa) was less than that of the Native group (38.65 ± 8.7 MPa) but with no significant difference (0.1 > *P* > 0.05). Furthermore, there was no significant difference of the Young’s moduli in compression between the three groups (109.5 ± 53.7 MPa for the Xeno group, 80.9 ± 52.9 MPa for the Allo group and 123.4 ± 98.0 MPa for the Native group, *P* > 0.05).

In terms of the creep test, the compressive creep at 1^st^ minute of the Xeno group was significantly less than that of the Native group (*P* = 0.03033). The tensile creep at 1^st^ minute (*P* = 0.043) and 10^th^ minute (*P* = 0.07) of the Allo group was significantly greater than those of the Native group. No significant difference was found in any of the creep test results between the Allo group and the Xeno group.

### Chondroprotection evaluation

**Figure 8:**
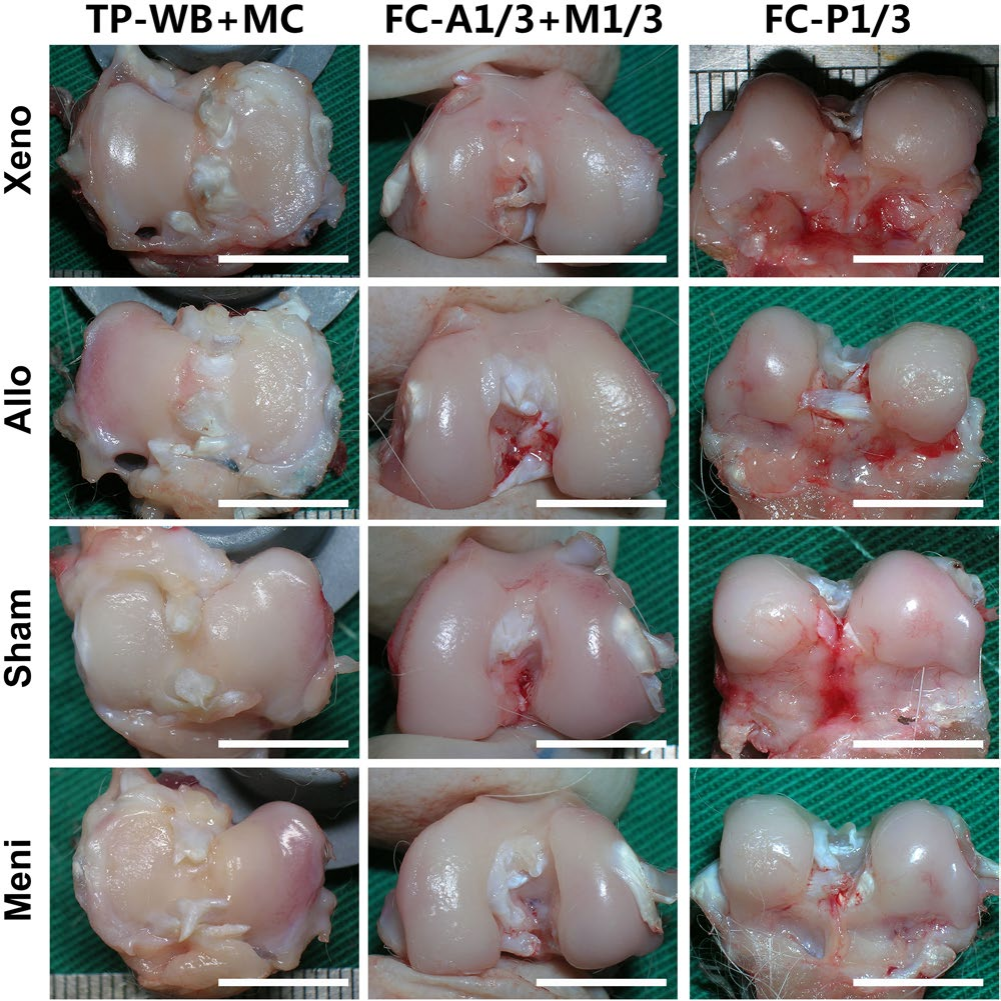
Macroscopic observations of joints at one year after operation. Compared with Sham group, various degrees of cartilage damage were observed in the other operated joints, especially in the Meni group. Scale bars = 10 mm.

According to the macroscopic evaluation of joints (Fig. 8), various degrees of cartilage damage were observed in all the transplantation and meniscectomy joints while no degradation was shown in Sham group.

**Figure 9:**
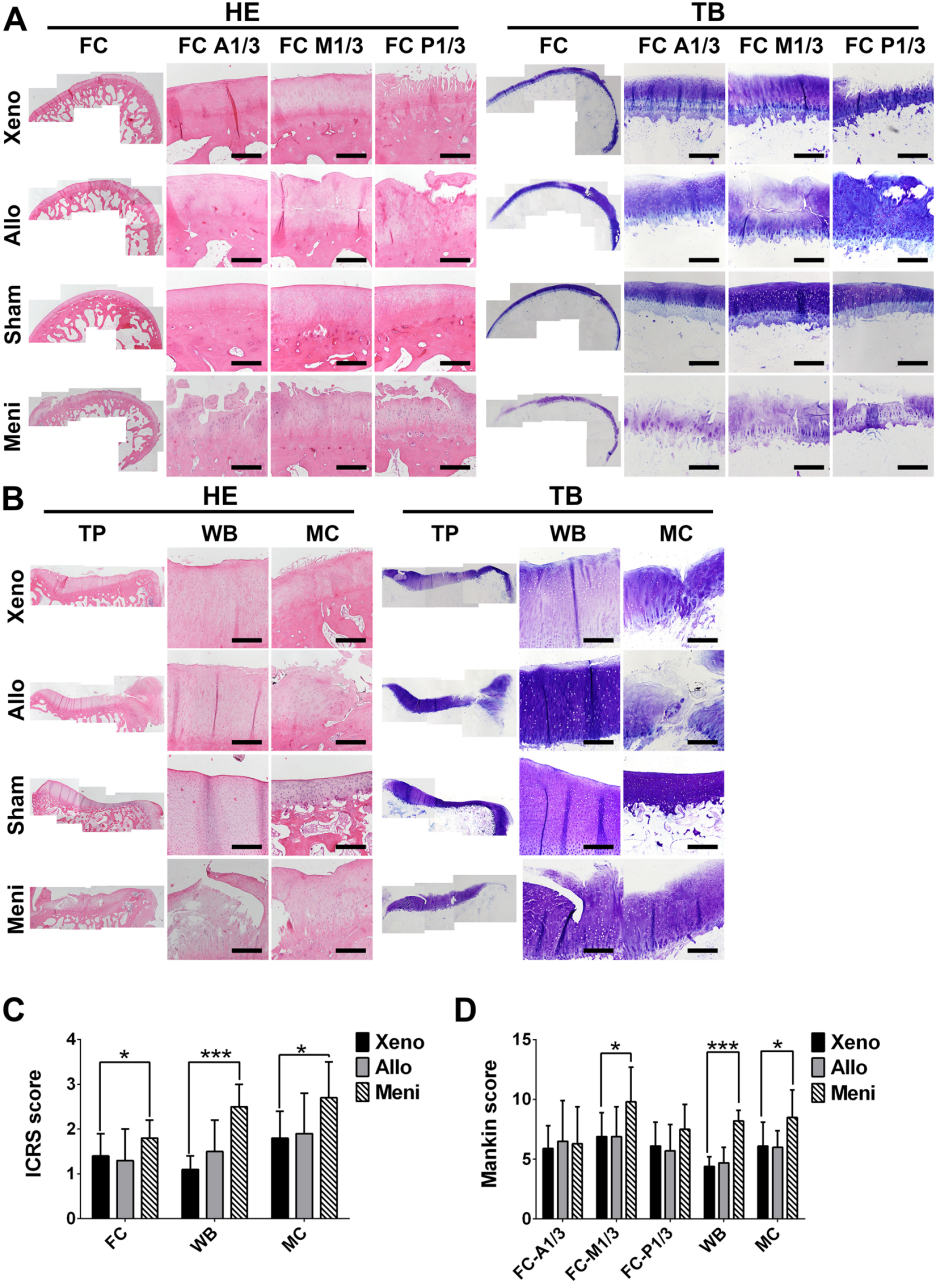
H&E and TB staining of articular cartilage surfaces in the femoral condyles (FC) (**A**) and tibial plateau (TP) (**B**). Scale bars = 100 μm. As indicated by International Cartilage Repair Society (ICRS) (**C**) and Mankin scores (**D**), the Xeno group presented lower cartilage degeneration in both the femur and tibia compared with Meni group (*P < 0.05, ***P < 0.001).

As shown in hematoxylin and eosin (H&E) and TB staining of articular cartilage surfaces in the femoral condyles (FC) and tibial plateau (TP) (Fig. 9A,B), complete disorganization of the cartilage and severe reduction of TB staining were revealed in the Meni group. The sections of both the Xeno and the Allo groups revealed clefts to the transitional or radial zone with slighter reduction of TB staining in FC, and the surface irregularities were also displayed in TP. According to the International Cartilage Repair Society (ICRS) and Mankin scores (Fig. 9C,D), the meniscus covered (MC) regions of the Xeno group showed significantly lower scores than those of the Meni group at postoperative 1 year (*P* < 0.05). The Mankin score at the FC-M1/3 of the Xeno group was significantly lower than that of the Meni group (*P* = 0.001). Although both of the Xeno and the Allo groups showed more serious joint degeneration compared to the Sham group (*P* < 0.05), there was no significant difference between the two groups.

## Discussion

The purpose of the present study was to evaluate the RDF-X as a substitute for the meniscus allograft. To our knowledge, this is the first study to examine the viability and the chondroprotective effects of xenogenic menisci in a one-year rabbit model. Throughout the relative long-term evaluation, similar tissue regeneration and cartilage protection were confirmed in the Xeno group compared to the Allo group. So far, no biologically derived or synthetic graft could completely prevent the potential damage of the post-meniscectomy joints. As the tissue closest to the native meniscus other than the allograft, the treated xenogeneic meniscus provided the extracellular matrix promoting cell incorporation and biomechanical support after transplantation. The positive results indicated that the RDF-X might be an alternative for meniscal replacement.

As in our previous report of the porcine RDF-X transplantation for 24 weeks, the treated xenogeneic meniscal tissue could heal the synovium with tissue regeneration and delay the degeneration of articular cartilage in the short-term^15^. However, some authors have noted that those grafts transplanted longer than 24 weeks usually led to construct ruptures and joint deterioration^16^. The main factor for those failures is the inadequate strength of the implants leading to long-term degeneration. Thus the rabbit model with a 1-year follow-up of the present study is useful for evaluating articular changes after meniscal transplantation in a longer term. In addition, the degenerative changes of articular cartilage in rabbit knees progress at a faster rate than in larger animals or humans^17^ and the present results could be a reference for the long-term outcome in the future clinical use.

In the present study, the RDF-X showed similar mechanical properties to the native meniscus in the range of 75–150 Mpa^18^. In spite of no significant difference in the tensile modulus between the three groups, a higher value of tension strength was observed in native menisci. This might be due to the fact that the regenerated menisci of the Xeno group contained fewer Col I, which directly contributed to resistance in tension loading^19^. These results might also explain the higher rate of meniscus tears in the Xeno group as showed in Table 1 and the ultimate tensile stress of both the Xeno and the Allo groups were significantly less than that of the Native group. The relatively poor tensile strength of the graft might be due to the treatment including radiation and deep freezing, which have been confirmed to destroy the collagen structure and lead to the reduction of the mechanical strength. Furthermore, the compressive modulus of the Xeno group was significantly more than that of the Allo group and the source location of the xenograft might be the cause. According to the previous study, the meniscus is structurally and functionally heterogeneous^20^. The xenograft of the present study was extracted from the inner 1/3 part of the pig meniscus consisting more collagen II and GAG, which showed higher compressive modulus compared to the outer part. It should be also noted that the compression testing was performed to the transected part, which only presented the mechanical strength of the tissue itself, instead of the biomechanical performance of the total meniscus. As for the meniscus works through tensile strength pulling from the anterior and posterior horns, the biomechanical testing with the total meniscus *in situ* should be studied in the future.

An early rejection was detected at week 2 in the blood serum and the result was similar to the previous xenogenic chondrocytes transplantation^21^. Despite that the radiation and the deep freezing weakened the immunogenicity of the xenogeneic meniscal tissue, the residual fragments of the dead cells still present in the xenograft. Thus the application of the xenograft caused the synovitis due to the release of the xenogeneic collagen and cell particles in the knee joint and the blood. The molecular ranged 60–80 kD but the antigen was still unknown, which might need further studies including immunoprecipitation. In addition, as we have reported previously, a large number of lymphocytes infiltration were observed around the xenograft at week 6. Those results indicated that both of the humoral immunity and the cellular immunity played a certain role in the early stage after the transplantation of the xenograft. At week 12, most of the xenografts were still surrounded by lymphocytes but with a much smaller number at week 24^15^. At postoperative 1 year, normal synovial cells surrounded all the xenograft except for two menisci with a small amount of lymphocyte infiltration. The immunological rejection seemed to have no significant effect on the long-term viability of the xenograft. However, there were three menisci of the group RDF-X with partial tears and rough surface and collagen collapse was detected in histological evaluation. Those might be the effect of the chronic rejection, which could not be detected by our presented methods. In spite of the relatively low immunogenity of the cartilage, the meniscus or the ligament, decellularization would be still necessary before the xenogenic extracellular matrix was used before transplantation.

Another interesting finding is that there was difference in the mode of the cell infiltration and tissue regeneration between the Xeno group and the Allo group. The cell incorporation were observed only at the upper and lower surface and the inner 1/3 part of the new regenerated meniscus in the Xeno group while most of the cell infiltration concentrated in the outer 1/3 part of the graft in the Allo group. Those results indicated that the regeneration of the RDF-X was realized by synovium enfolding other than gradually cell incorporation from the peripheral synovium. The regeneration mode of the RDF-X might be mostly related to the immune rejection to the tissue with heterogeneous origin. In addition, the phenotype of the regenerated cells of the Xeno group demonstrated more approximated hyaline chondrocyte surrounded by a little more TB staining and Col II but with fewer Col I and III, which could be the effect of the compressive stimulus from the femur and the tibia differentiating the synovial cells or the stem cells in the enfolding synovium into the chondrocyte^22^.

The main limitation in this study is that the implant was only obtained from the inner region of meniscal tissue without peripheral synovium, which contains more immunogens. Due to the heterogeneous characters of meniscus, the inner region contained large amounts of Col II and glycosaminoglycans with little immunogenicity. Therefore the initial biomechanical properties was not equivalent to those of native menisci. Thus further studies might research different regions of menisci for xenogenic grafts. Secondly, the rabbit-anti-pig antibody detection was only performed in the early 6 weeks. Although the antibody was not detected in the 4–6 week, reappearence is possible in the later stages. Thirdly, surgeries were performed for both knees for all the rabbits for the sake of reducing animal use. There might due to the impact of sham and total meniscectomy on the xenograft and allograft, respectively.

## Conclusions

The RDF-X showed similar results to allogenic meniscus at 1-year follow-up. However, some problems such as the potential immunological rejection and the shape matching remain to be unsolved before clinical application.

## Materials and Methods

All animal experiments were complied with the “Guide for the Care and Use of Laboratory Animals” published by the National Academy Press (NIH Publication No. 85–23, revised 1996) and approved by the Animal Care and Use Committee of Peking University.

### Harvest and preparation of the xenogenic meniscal tissues

10 large white pigs were obtained from other unrelated studies within 24 hours of animal sacrifice, and their menisci were removed for use as the source of xenografts. The pig meniscus was washed in phosphate-buffered saline (PBS, Sigma Diagnostics, St Louis, MO) to remove excess blood and the inner one-third part of the meniscus tissue was shaped into wedge-shaped semilunar discs using the removed rabbit medial meniscus as template. The allograft menisci were also harvested from the xenograft group after total medial meniscectomy. Then the shaped xenogenic and allogenic meniscal tissues were packaged separately and treated with 25 k Gray gamma irradiation and deeply frozen (−80 °C) for 6 to 14 days.

### ***In vitro*** assessment of the xenogenic meniscal tissues

#### Light Microscopy

The treated xenogenic meniscal tissues was fixed in 10% (v/v) neutral buffered formalin (Sigma Diagnostics, St Louis, MO) and then dehydrated in alcohol and embedded in paraffin. Five-micrometer-thick sections were cut and then stained with H&E and TB (as a label for proteoglycans). Besides, sections were treated with IHC procedure labeling Col I and II as previous described^23,24^.

#### Scanning Electron Microscopy

Specimens were fixed in a solution of 1% (w/v) osmium tetroxide and 1.5% (w/v) potassium ferrocyanide for 3 h. Slices were washed in pH 7.2 PBS, dehydrated in ascending grades of ethanol and subjected to critical-point drying in CO_2_. Dried slices were mounted on standard stubs, gold-coated in a sputter coater and then observed on a SEM microscope (SEM100-CX II, Japan).

### ***In vivo*** assessment of the xenogenic meniscal tissues

#### Study design

20 skeletally mature male New Zealand White rabbits weighing between 2.0 and 2.5 kg were included in this study. Total medial meniscectomy were performed for the right knees of all the rabbits. To ensure the uniform time interval and avoid secondary operation, all the allografts were from an unrelated study with the same treatment procedure as the xenograft. Both of the treated allograft and the xenograft were prepared before the operations and were transplanted immediately after meniscectomy. Ten right knees received the treated xenogenic meniscal tissues (Xeno group) and 10 others allograft menisci (Allo group). The left knees of the Allo group underwent total medial meniscectomy without implantation of a graft (Meni group). As a control, the left knees of the Xeno group underwent sham operation involving exposure of the medial joint and close in layers (Sham group). All the rabbits were sacrificed at postoperative 1 year.

#### Surgical procedures

No immunosuppressive agents were used in this study. Intramuscular penicillin (400 thousand units) was administered preoperatively as antibiotic prophylaxis. The rabbits were anesthetized with intravenous urethane (1 g/kg). The surgical procedure was performed as previously reported^24^. Briefly, the right knee was approached through a medial parapatellar incision. Medial meniscus was resected sharply along the periphery and detached from their anterior and posterior junction. Care was taken not to injure the medial collateral ligament which was important for the postoperative stability of knee joints. The treated xenogenic meniscal tissue or the meniscus allograft was thawed by immersion in sterile saline and then sutured to the adjacent synovium with non-resorbable 5-0 sutures (Ethicon, Johnson & Johnson, Amersfoort, Netherlands). The extracapsular knotting technique was used for the posterior horn. The anterior and posterior horns of the graft were reattached to the appropriate ligamentous structures. The capsule, periarticular tissues, and skin were closed with Vicryl 3-0 sutures (Ethicon, Johnson & Johnson). For the Meni group, only total resection of the medial meniscus was performed. For the Sham group, the operation was performed on the medial compartment using the same approach required for the meniscectomy procedure without operating on the meniscus. After the operation, the animals were immediately allowed to have movement and weight-bearing activity and were not restricted in any way. The antibiotic prophylaxis was continued for 3 days.

### Evaluation of initial immunological rejection

At postoperative week 2, week 4 and week 6, 1 ml blood serum was extracted from the rabbit of each group. Meanwhile, 1 ml PBS was infiltrated into the transplanted knee joint and was then extracted as the dilution of synovial fluid. Both of the blood serum and the dilution of synovial fluid was treated with Western-blot program to detect the Rabbit-anti-Pig antibody.

The shredded pig meniscal tissue was lysed in buffer for 30 min. Samples of the lysate were resolved on SDS-PAGE 12% (v/v) gels. For Western blot assays, the separated protein bands were electro-blotted onto nitrocellulose membranes, at a constant current of 250 mA in transbuffer (50 mM Tris, pH 8.0, containing 0.192 M glycine and 20% (v/v) methanol), using a Bio-Rad Trans-Blot Cell. The strips were incubated for 1 hr at room temperature in blocking buffer (TBS containing 5% (w/v) nonfat milk), followed by an overnight incubation at 4 °C with constant agitation in 1:40 dilution of serum and dilution of synovial fluid in blocking buffer. After 3 washes with TBS containing 0.05% (v/v) Tween 20, strips were incubated for 1 hr with appropriate HRP-conjugated anti-immunoglobulin reagents and the antigen-antibody complexes visualized using the ECL detection system as recommended by the manufacturer (Applygen Technologies Inc., Beijing, China). Data was recorded with Bio-Rad GelDoc2000.

### Evaluation of meniscus

#### Gross evaluation

The joints were dissected with the femur separated from the tibia and the meniscus left attached to the tibia plateaus. Photographs were taken (Nikon 4600, Nikon Photo Products Inc, Tokyo, Japan) of the tibial plateau and of the exposed femoral condyles. The menisci were grossly evaluated by the integration, implant position, horn position, shape, tears, surface, size, tissue and the synovium reaction^25^. Each parameter was scored 1 to 3 based on the situation of the implants.

#### Histological evaluation

**Figure 10:**
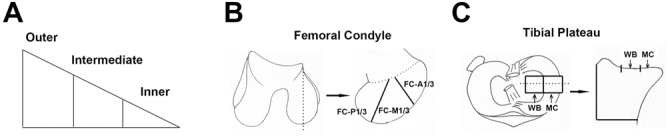
Regional division of meniscal tissue and osteochondral specimens. Meniscus was devided into outer, intermediate and inner regions (**A**). Medial FC were divided evenly into anterior (FC-A1/3), middle (FC-M1/3) and posterior (FC-P1/3) third regions (**B**). The medial TP was divided into weight bearing (WB) regions and meniscus covered (MC) regions (**C**).

The menisci were separated from joints and treated with the above-mentioned routine procedure. Then, five-micrometer-thick sections were sliced and then stained with H&E, TB and Col II. Besides, sections were treated with PR staining to observe collagen type I and III. Meniscus was cut to produce blocks showing their wedge-shaped profile, which were divided into outer, intermediate and inner regions of the regenerated menisci in histological sections (Fig. 10A). The sections were evaluated according to the meniscus histology scoring system^16^. The number of integrated cells and the TB metachromasia in each zone were semi-quantitatively evaluated separately. Cell number: 0-no cell ingrowth, 1-very small amount of cell ingrowth, 2- moderate amout of cell ingrowth, 3-large number cell ingrowth, 4- normal amount of cell growth. TB metachromasia: 0- no, 1-mild, 2-obvious. All the sections were evaluated by 3 experienced researchers and the mean score was attained as the final results.

#### Ultrastructure evaluation

The menisci of the Allo group and the Xeno group and the normal rabbit tissue of the Sham group were treated with the same SEM procedure as mentioned above.

#### Biomechanical properties evaluation

The tensile and the compressive samples were cut into rectangular shapes. The tensile testing samples were about 3 × 6 × 1 mm^3^ and the compressive samples were about 2 × 2 × 1 mm^3^. The biomechanical properties of specimens were tested by a material testing machine (AG-IS, Shimadzu, Japan) with a 50 N load^23^. The working temperature is about 23 °C and the test specimens were kept hydrated using 0.9% (w/v) NaCl solution. For the tensile testing, the tissue was preconditioned to a maximum displacement of 0.5 mm at a displacement rate of 0.5 mm/min for 8 cycles. The sample was tested to ultimate failure at a rate of 0.5 mm/min^26^. For the compressive testing, the tissue was loaded at a displacement rate of 0.1 mm/min with a maximum force of 10 N for 8 cycles. The elastic modulus was analyzed from the linear portion of the stress-strain curve, and only used the date of 5 cycles after 3 preconditioned cycles^27^.

### Evaluation of chondroprotection

Three researchers who were blinded to the experimental groups independently evaluated the cartilage macroscopically of the femur and tibia according to the criteria of the ICRS cartilage injury classification^28^. The cartilage of the medial femoral condyle and the tibial plateau were analyzed and the medial tibial plateau was divided into the WB and MC regions (Fig. 10C). Each region was evaluated once by one researcher. The mean score of the researchers was then taken for the final evaluation.

The osteochondral specimens were decalcified in 10% (w/v) EDTA (Titriplex III, Merck, Darmstadt, Germany) for 3 weeks. When the decalcification was completed, the osteochondral specimens were sectioned in the coronal plane at the midpoint of the tibial plateau in the axial plane at the midpoint of the medial femoral condyle (Fig. 10B,C). Histological sections of medial FC were divided evenly into anterior (FC-A1/3), middle (FC-M1/3) and posterior (FC-P1/3) third regions. The sections of the medial tibial plateaus were divided into WB and MC regions. Each region of the histologic sections was graded with the Mankin grading system for hyaline cartilage degeneration^29^. All histological sections were scored by three attending pathologists blinded to the procedures performed. The mean score of both investigators was used for the final evaluation.

### Statistical analysis

The nonparametric test was used for the skewness data, unknown distribution data or data with heterogeneity of variance. The Mann-Whitney rank-sum test was used to compare macroscopic and histological scores between different groups and regions. The T-test was used for the mechanical evaluation. All statistical analyses were completed with SPSS 11.5 software program. Statistical significance was set at *P* < 0.05.
